# Matrisome analysis of intrahepatic cholangiocarcinoma unveils a peculiar cancer-associated extracellular matrix structure

**DOI:** 10.1186/s12014-019-9257-x

**Published:** 2019-10-30

**Authors:** Guido Carpino, Diletta Overi, Fabio Melandro, Alessio Grimaldi, Vincenzo Cardinale, Sabina Di Matteo, Gianluca Mennini, Massimo Rossi, Domenico Alvaro, Vincenzo Barnaba, Eugenio Gaudio, Carmine Mancone

**Affiliations:** 1 Division of Health Sciences, Department of Movement, Human and Health Sciences, University of Rome “Foro Italico”, Piazza Lauro de Bosis 6, 00135 Rome, Italy; 2 Department of Anatomical, Histological, Forensic Medicine and Orthopedics Sciences, Sapienza University of Rome, Via Borelli 50, 00161 Rome, Italy; 3 Department of General Surgery and Organ Transplantation “P. Stefanini”, Sapienza University of Rome, Viale del Policlinico 151, 00161 Rome, Italy; 4 Department of Internal Medicine and Medical Specialties, Sapienza University of Rome, Viale del Policlinico 151, 00161 Rome, Italy; 5 Department of Medico-Surgical Sciences and Biotechnologies, Sapienza University of Rome, Corso della Repubblica 79, 04100 Latina, Italy; 6 Department of Translational and Precision Medicine, Sapienza University of Rome, Viale del Policlinico 151, 00161 Rome, Italy; 7 Department of Molecular Medicine, Sapienza University of Rome, Viale Regina Elena 291, 00161 Rome, Italy

**Keywords:** Matrisome, Cholangiocarcinoma, Desmoplastic stroma, Collagen type III, COL3A1

## Abstract

**Background:**

Intrahepatic cholangiocarcinoma (iCCA) is a malignancy that arises from the intrahepatic biliary tree, showing high mortality rates due to its late clinical presentation and limited treatment options. iCCA is characterized by a dense, reactive desmoplastic stroma marked by a dramatic accumulation of extracellular matrix (ECM). Although recent results strongly suggest a relationship between increasing desmoplastic stroma and the enhanced malignant behaviour of iCCA, the importance of ECM proteins in the pathogenesis of iCCA still have to be addressed.

**Methods:**

iCCA ECM fibrillar structural organization was characterized by histological analysis. ECM proteome profiles from decellularized iCCA and surrounding noncancerous tissues were analysed by nLC coupled to MALDI-TOF/TOF analysis.

**Results:**

iCCA tissues displayed high levels of collagen fibers and low abundance of reticular and elastic fibers, suggesting stiffness and loss of polarity. The ECM proteome profiles of iCCA samples, when compared to those obtained from the surrounding noncancerous tissues showed a dismantling of the basement membrane, a reduced angiogenesis and a downregulation of oncosuppressive activity. In particular, we focused on the effects of the overexpression of collagen type III alpha 1 chain (COL3A1) in iCCA, thus providing evidences that COL3A1 promotes iCCA cells migration and is a component of tumor-associated aligned collagen.

**Conclusions:**

Overall, this study contributes to the understanding of molecular basis underlying desmoplasia in iCCA and indicates the type III collagen as a promising therapeutic target.

## Background

Intrahepatic cholangiocarcinoma (iCCA) is a highly lethal primary liver cancer that originates from the intrahepatic biliary tree [1]. iCCA represents the second most common hepatic malignancy after hepatocellular carcinoma (HCC), accounting for 10% of all primary liver malignant neoplasms. However, its incidence and mortality are increasing worldwide, representing a significant health problem [2]. The clinical management of patients with iCCA is still a challenge. In fact, late and silent clinical manifestations, high recurrence rate after hepatic resection, and limitations in treatment options in patients with advanced metastatic iCCA lead to a very high levels of mortality [3]. To date, the surgical resection is considered as the only strategy with the potential to cure. However, only patients with a localized disease may undergo surgery and, therefore an early and specific diagnosis of iCCA is mandatory for resectability. At present, the unknown aetiology of iCCA, its paucicellular nature and the difficulty in distinguishing it from other malignant and benignant hepatic masses limited both the development of therapeutic targets as well as the identification of specific markers useful for the early cancer detection [4].

iCCA is characterized by striking and diffuse desmoplastic, hypovascularized stroma [5]. During iCCA carcinogenesis, the desmoplastic reaction originates by the accumulation of many α-smooth muscle actin (α-SMA)-positive cancer associated fibroblasts (CAFs) which in turn lead to an increased of an aberrant extracellular matrix (ECM) production [6, 7]. This fibrogenic response causes histopathological lesions that surround neoplastic bile ducts where CAFs promote tumor progression and invasiveness via ECM molecular components [8]. In the last years, growing evidences highlighted the importance of ECM proteins in the pathogenesis of iCCA. Among them, periostin (POSTN), a matricellular protein overexpressed in iCCA, has been found to correlate with shorted survival times of patients [9]. Knockdown of the POSTN receptor was able to reduce the POSTN-mediated tumor proliferation and invasion [10]. Similarly, stromal cell-derived factor-1 (CXCL12), an ECM secreted factor, has been found to play a role in promoting cholangiocarcinoma cell migration and invasion [11]. ECM degradation and remodelling promoted by matrix metalloproteinases (MMPs) are required for fibrogenesis and desmoplastic tumor progression. In particular, MMP1, 2, 3 and 9 have been found to be overexpressed in cholangiocarcinoma [12]. Moreover, the tissue inhibitor of metalloproteinases 3 (TIMP3) has been observed to be downregulated [12, 13]. Dysregulations of these ECM regulators are usually associated with primary liver tumors [13]. In the wake of these data, it is conceivable that ECM proteins could be the targets of new therapeutic bullets as well as potential biomarkers of the disease. To achieve these goals, a fine characterization of the ECM proteome, or “matrisome,” is desirable. In this paper, we characterized the iCCA ECM composition with respect to the non-cancerous liver tissue (NCT), unveiling a peculiar iCCA matrisome profile. We focused on the collagen type III alpha 1 chain (COL3A1) stromal overexpression, demonstrating its involvement in iCCA cells migration and in the tumor-associated collagen re-organization.

## Methods

### Liver tissue decellularization, protein digestion, and peptide purification

For each patient, 10 mg of both iCCA and NCT tissues from surgery resections underwent tissue decellularization as previously described [14]. Briefly, plasma proteins removal from tissues was performed by overnight shaking (600 RPM) in Washing Buffer (0.5 M NaCl, 10 mM Tris Base pH 7.5, 1× protease inhibitor) at 4 °C. After centrifugation at 13,000 RPM for 1 min, the supernatants (plasma proteins) were stored at − 80 °C. Pellets were washed twice with Decellularization Buffer (DB) (1% SDS in PBS, 1× protease inhibitor) and incubated overnight in DB at room temperature, shaking at 800 RPM. On the next day, the supernatants were removed and stored at − 80 °C and fresh DB was added to the tissues. This process was repeated until the tissues were completely decellularized. Decellularized tissues were washed twice with deionized water and incubated with 80% acetone for 90 min to remove residual SDS. After centrifugation at 12,000 RPM for 15 min at 4 °C the supernatants were discarded, and the ECM scaffolds were washed twice with PBS. For proteomics analysis, ECM scaffolds obtained from the decellularization of 10 mg of specimen were resuspended in 40 μL of NH_4_HCO_3_ 50 mM, urea 2 M and digested with 5 μL of trypsin solution [0.2 μg/μL] overnight at 37 °C. Scaffolds were then centrifuged at 12,000 RPM for 15 min at 4 °C and the obtained ECM peptides (supernatant) were reduced in 10 mM Dithiothreitol (DTT) at 56 °C for 30 min. Alkylations were performed in 55 mM Iodoacetamide (IAM) in the dark at room temperature for 20 min. Peptides were then desalted and filtered through a C18 microcolumn ZipTip™ (MercK-Millipore, Darmstadt, Germany, cod. ZTC185096) and eluted from the C18 bed using 10 μL of 80% acetonitrile/0.1% trifluoroacetic acid. The organic component was removed by evaporation in a vacuum centrifuge and peptides were resuspended in 7 μL 2.5% acetonitrile/0.1% trifluoroacetic acid for the subsequent nano-liquid chromatography (nanoLC) analysis. All reagents were from Sigma Aldrich except where expressly indicated.

### NanoLC analysis and mass spectrometry analysis

Samples were analysed by nano-LC coupled to MALDI-TOF/TOF mass spectrometry as previously described [15]. In brief, An UltiMate 3000 RSLC (rapid separation liquid chromatography) nano-LC system (ThermoFisher Scientific, MA—USA) equipped with an integrated nanoflow manager and microvacuum degasser was used for peptide separation. The peptides (7 μL) were loaded onto a 75 μm I.D. NanoSeries C18 column (ThermoFisher, P/N 164,534) for multistep gradient elution (eluent A 0.05% TFA; eluent B 0.04% TFA in 80% ACN) from 5 to 20% eluent B within 10 min, from 20 to 50% eluent B within 45 min and for further 5 min from 50 to 90% eluent B with a constant flow of 0.3 μL/min. After 5 min, the eluted sample fractions were continuously diluted with 1.2 μL/min a-cyano-4-hydroxycinnamic acid (CHCA) and spotted onto a MALDI target using a HTC-xt spotter (PAL SYSTEM) with an interval of 20 s resulting in a total of 168 fractions for each sample injection. Mass Spectrometry Analysis MALDI-TOF–MS spectra were acquired using a 5800 MALDI TOF/TOF Analyzer (Sciex, Ontario—Canada). The spectra were acquired in the positive reflector mode by 20 subspectral accumulations (each consisting of 50 laser shots) in an 800–4000 mass range, focus mass 2100 Da, using a 355 nm Nb:YAG laser with a 20 kV acceleration voltage. Peak labeling was automatically done by 4000 Series Explorer software Version 4.1.0 (Sciex) without any kind of smoothing of peaks or baseline, considering only peaks that exceeded a signal-to noise ratio of 10 (local noise window 200 m/z) and a half maximal width of 2.9 bins. Calibration was performed using default calibration originated by five standard spots (Mass Standards kit for Calibration P/N 4333604). Only MS/MS spectra of preselected peaks were integrated over 1000 laser shots in the 1 kV positive ion mode with the metastable suppressor turned on. Air at the medium gas pressure setting (1.25 × 10^−6^ Torr) was used as the collision gas in the CID off mode. After smoothing and baseline subtractions, spectra were generated automatically by 4000 Series Explorer software. MS and MS/MS spectra were processed by ProteinPilot Software 4.5 (Sciex) which acts as an interface between the Oracle database containing raw spectra and a local copy of the MASCOT search engine (Version 2.1, Matrix Science, Ltd.). The Paragon algorithm of ProteinPilot Software 4.5 was used with identification as the Sample Type, iodacetamide as cysteine alkylation, with the search option “biological modifications” checked, and trypsin as the selected enzyme (one missed cleavage site was allowed). MS/MS protein identification was performed against the human Uniprot/SwissProt database (released on 07/06/2016) using a confidence threshold of 95% (Proteinpilot Unused score ≥ 1.31). The monoisotopic precursor ion tolerance was set to 0.12 Da and the MS/MS ion tolerance to 0.3 Da. The minimum required peptide length was set to 6 amino acids; two peptides were required for protein identification. A decoy search was carried out in order to estimate the false discovery rate (FDR). Only peptides identified with a FDR < 5% were assembled into identified proteins. Protein identifications (at a specified critical FDR of 5%) were automatically generated by the Paragon Algorithm™ by measuring all the peptide evidences for a protein in a single LC run. For each sample, an estimate of relative abundance (protein score) was obtained by dividing the total unique peptides count for the length of the protein in amino acids.

### Histological analyses

For each patient, specimens included iCCA and NCT; samples were collected before and after the decellularization procedure, fixed in 10% buffered formaldehyde, and embedded in paraffin. From each specimen, 3-μm sections were obtained and processed for routine histo-morphological stains: hematoxylin-eosin (H&E); Sirius Red (SR) counterstained with hematoxylin or Fast Green for collagen fibers; Weigert’s stain (Bio-Optica Milano S.p.a., Milano, Italy, Code: 04-053812) for elastic fibers; Gomori’s silver impregnation (Bio-Optica Milano S.p.a., Code: 04-040801) for reticular fibers. Specimens were scanned by a digital scanner (Aperio Scanscope CS System, Aperio Digital Pathology, Leica Biosystems, Milan, Italy) and processed by ImageScope. For each stain, image analysis software was set to recognize stained fibers, and was ran on the entire section; specimens displayed at least 5 non-overlapping field at 20× magnification. The area occupied by fibers was calculated and expressed as percentage with respect to the total area of the section. For immunohistochemistry, endogenous peroxidase activity was blocked by a 30-min incubation in 2.5% methanolic hydrogen peroxide. Antigens were retrieved, as indicated by the vendor, by applying Proteinase K (Dako, Glostrup, Denmark, code S3020) for 10 min at room temperature. Sections were then incubated overnight at 4 °C with primary antibody against COL3A1 (Santa Cruz Biotechnology, Inc, Dallas, TX, USA; Code: sc-8781), proteoglycan (PRG)2 (Millipore, Burlington, MA, USA; code: MABT400); PRG4 (ThermoFischer Scientific, Waltham, MA, USA; code: PA5-30130). Samples were then rinsed twice with phosphate buffered saline (PBS) for 5 min, incubated for 20 min at room temperature (RT) with secondary biotinylated antibody, and then with Streptavidin–horseradish peroxidase (LSAB+, Dako, Glostrup, Denmark code K0690). Diaminobenzidine (Dako, Glostrup, Denmark code K3468) was used as substrate, and sections were counterstained with hematoxylin. Negative controls (the primary antibody was replaced with pre-immune serum) were also included. Slides were scanned and processed by ImageScope; then, an image analysis algorithm was used to calculate the percentage of stained area in at least 5 non-overlapping fields at 20×, and a semi-quantitative (SQ) scoring system was applied (0 = < 10%; 1 = 10–30%; 2 = 31–50%; 3 = > 50%). Data (Median) are visualized as a heat map.

### iCCA primary cell cultures and migration assays

Primary cell cultures were prepared from specimens of human intrahepatic iCCA obtained from surgical resection of patients and characterised as previously described [16–18]. The use of human materials was approved by Institutional Review Board and the research protocol was approved by the Ethics Committees of the Policlinico Umberto I Hospital. Primary iCCA cell cultures were maintained in specific medium, H69, in a humidified atmosphere (37 °C, 5% CO_2_) as described in [17, 18]. In the present study, we used iCCA primary cell cultured after 40 passages from isolation. Cell proliferation was evaluated by MTS assay (CellTiter 96^®^ AQueous MTS Reagent Powder, Promega). Collagen I (Col I, Sigma, C5483) and Collagen III (Col III, Millipore, CC054) were diluted in acetic acid 0.02 M in DPBS (Gibco, 14190250) at the final concentration of 250 µg/mL, and 50 µL per well were used for coated each well of 96-well plate. After 24 h of incubation at room temperature, wells were washed with 100 µl of DPBS for 20 min for three times. Subsequently, 104 cells in 100 µL of H69 per well were seeded, and polystyrene surface was used as control. The H69 medium was replaced with fresh medium every 2 days. After 72 h of cells seeding, MTS assay was performed as described in refs. [16–18]. To evaluate the migration capacity of iCCA primary cell line, the OrisTM Universal Cell Migration Assembly Kit (Platypus Technologies, CMAU101) was used. Col I (Sigma, C5483) and Col III (Millipore, CC054) were diluted in acetic acid 0.02 M in DPBS (Gibco, 14190250) at the final concentration of 250 µg/mL, and 50 µL were used for coated each well of OrisTM Universal Cell Migration Assembly Kit. After 24 h of incubation at room temperature each well was washed with 100 µL of DPBS 20 min for three times. According to product data sheet we removed the DPBS and populated the 96-well plate with Cell Seeding Stoppers. Subsequently, 100 µL of suspended cells (104 cells/well) were pipetted into each test well through one of the side ports of the Cell Seeding Stopper. The seeded plate containing the Seeding Stoppers was incubated at 37 °C in a humidified atmosphere of 5% CO_2_ for 24 h to permit cell attachment. After 24 h the Cell Seeding Stopper was removed. To remove any unattached cells, the H69 medium was removed, and wells were gently washed with 100 µL of sterile DPBS; at the end of the procedure, 100 µL of fresh H69 medium was added and changed every 2 days. The polystyrene surface and time equal to 0 (t = 0) were used as pre-migration controls for each condition. After 7 days, the cells were fixed and stained with 0.1% crystal violet in ethanol (Sigma-Aldrich). The cells were photographed by Nikon 50D Camera with 4× magnification. Pictures were analysed through Adobe Photoshop Software and the area covered by cells was quantified and is expressed as percentage of covered area (percentage of migration). The cells that migrated on polystyrene were considered as a positive control and their migration was considered equal to 100%.

### ct-FIRE and CurveAlign collagen architecture assessment

Total Collagen and COL3A1 fibers were respectively identified from SR and immunofluorescence images of patient tissues using the ct-FIRE software package (http://loci.wisc.edu/software/ctFIRE, v.1.3b). Specifically, the analysis parameters included: ‘CT-FIRE Fibers’ analysis method and set to ‘No Boundary’ boundary method; ‘Minimum length of free fibers’ to 20. Further analysis of fiber to fiber orientation was completed using the CurveAlign software package (http://loci.wisc.edu/software/curvealign, v.3.0b). All iCCA and NCT samples included in this study were examined (N = 9). For each sample, at least 5 non overlapping microscopic fields at 20× were acquired and analysed with the software package.

### Immunoblotting analysis

Soluble proteins from tissues were obtained by overnight shaking (600 RPM) in 0.5 M NaCl, 10 mM Tris Base pH 7.5, 1× protease inhibitor buffer at 4 °C. After centrifugation at 13,000 RPM for 1 min, the supernatants (soluble proteins) were collected and quantified. Samples (10 γg) were separated in NuPAGE™ 4–12% Bis–Tris Midi Protein Gels (Life technologies, Thermo Fisher Scientific, Waltham, MA, USA) by SDS-PAGE with MOPS running buffer (Life technologies) and electroblotted onto nitrocellulose (GE, Healthcare, Little Chalfont, UK) membranes. The blots were incubated with primary and secondary antibodies. The antibodies were revealed using Luminata™ Western HRP Chemiluminescence Substrates (Millipore Corporation, Billerica, MA 01821 U.S.A). To control for equal protein loading and transfer, the membranes were stained with Ponceau S solution (Sigma, St. Louis, MO, USA). The following antibody was used: anti-COL3A1 (Santa Cruz Biotechnology, Dallas, Texas, USA, sc-8781). The secondary anti-rabbit peroxidase-conjugated antibody was from Jackson ImmunoResearch (Cambridge House, UK). The chemiluminescent blots were imaged with the ChemiDocTM Touch Imaging System (Bio-Rad Laboratories, Hercules, CA, USA).

### Statistical analysis

Statistical differences were calculated using Student *t*-test. All tests are two-tailed, and a *p*-value of < 0.05 was considered as statistically significant. Wilcoxon test was used to compared paired samples (NCT versus iCCA) in histo-morphological and immunohistochemical analyses. Statistical test is indicated within figure legends. The analyses were performed using SPSS software v.23 (IBM, Milan, Italy).

## Results

### iCCA has an altered ECM fiber composition in respect to NCT

**Fig. 1:**
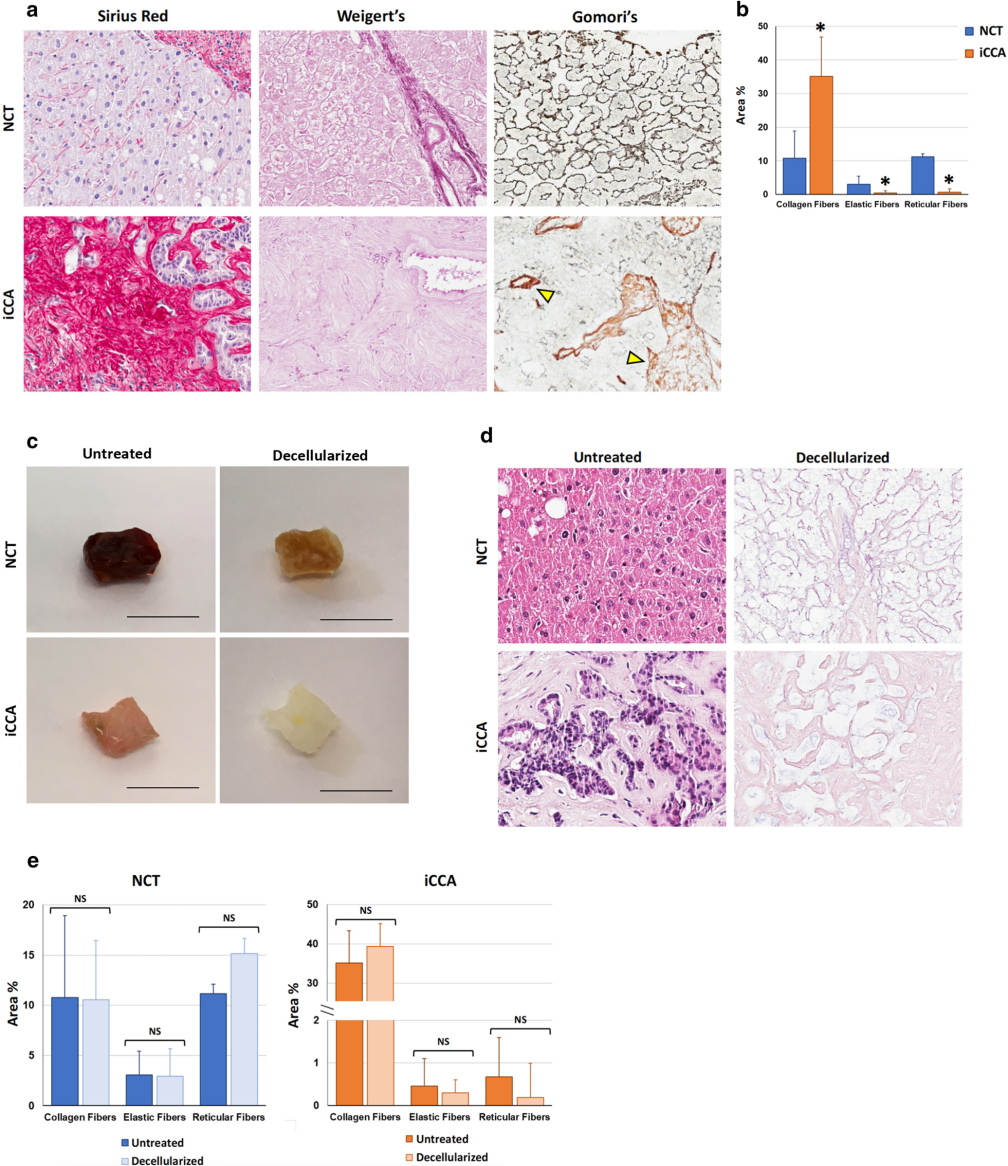
Collagen, elastic and reticular fibers’ composition analysis unveils altered ECM structure in iCCA tissues in respect to paired NCT (N = 9). **a** Representative images of NCT and iCCA histomorphological stains for collagen (Sirius Red, stained in magenta) elastic (Weigert’s, stained in purple) and reticular (Gomori’s silver impregnation, stained in black) fibers. Original Magnification (OM) = ×20. Arrowheads indicate collagen fibers (stained in brown). **b** Histograms shows area % of the sample occupied by tissue fibers. Data are expressed as mean ± standard deviation (SD). *p < 0.05 vs. paired NCT (Wilcoxon test). **c** Representative images of NCT and iCCA surgical specimens before and after the decellularization. Scale bars: 5 mm. **d** Hematoxylin and eosin stain on NCT and iCCA samples before and after decellularization. OM = ×20. **e** Histograms for area % of the samples occupied by tissue fibers in NCT and iCCA ECM composition before and after decellularization (paired samples). Analyses were performed on the entire section for each specimen (at least 5 fields at ×20 magnification). Data are expressed as mean ± SD. Wilcoxon test was applied. NS = *p *> 0.05

Tissue specimens were obtained from surgical resections of 9 patients (Additional file 1). We firstly characterized the iCCA and NCT ECM structures in relation to the collagen, elastic and reticular fibers composition (Fig. 1a, b). In iCCA samples, Sirius Red staining revealed a stroma rich in collagen fibers that surrounds malignant cells compared to the few collagen fibers observed in paired NCT samples (p < 0.05). On the contrary, Weigert’s staining and Gomori’s silver impregnation stains revealed, respectively, only traces of elastic and reticular fibers in cancerous tissues compared to paired NCT (p < 0.05), thus indicating a peculiar ECM architecture in the iCCA microenvironment.

Liver tissue decellularization is required for purifying the ECM in anticipation of the matrisome analysis [14, 19]. Therefore, since the cancerous ECM fiber pattern originates from a specific molecular profiling, we decellularized the iCCA and NCT tissues to assess if the isolated ECM scaffolds were suitable for a comparative proteomics analysis (Fig. 1c). As shown in Fig. 1d, H&E stain highlighted that no nuclei or cytoplasmic staining were observed in both NCT and iCCA tissues, thus showing highly purified ECM scaffolds. Most importantly, the analysis of fiber components indicated that the decellularization process did not affect the overall ECM structure in paired samples (Fig. 1e).

### Matrisome proteomics on decellularized tissues reveals a unique signature of ECM proteins in iCCA

**Fig. 2:**
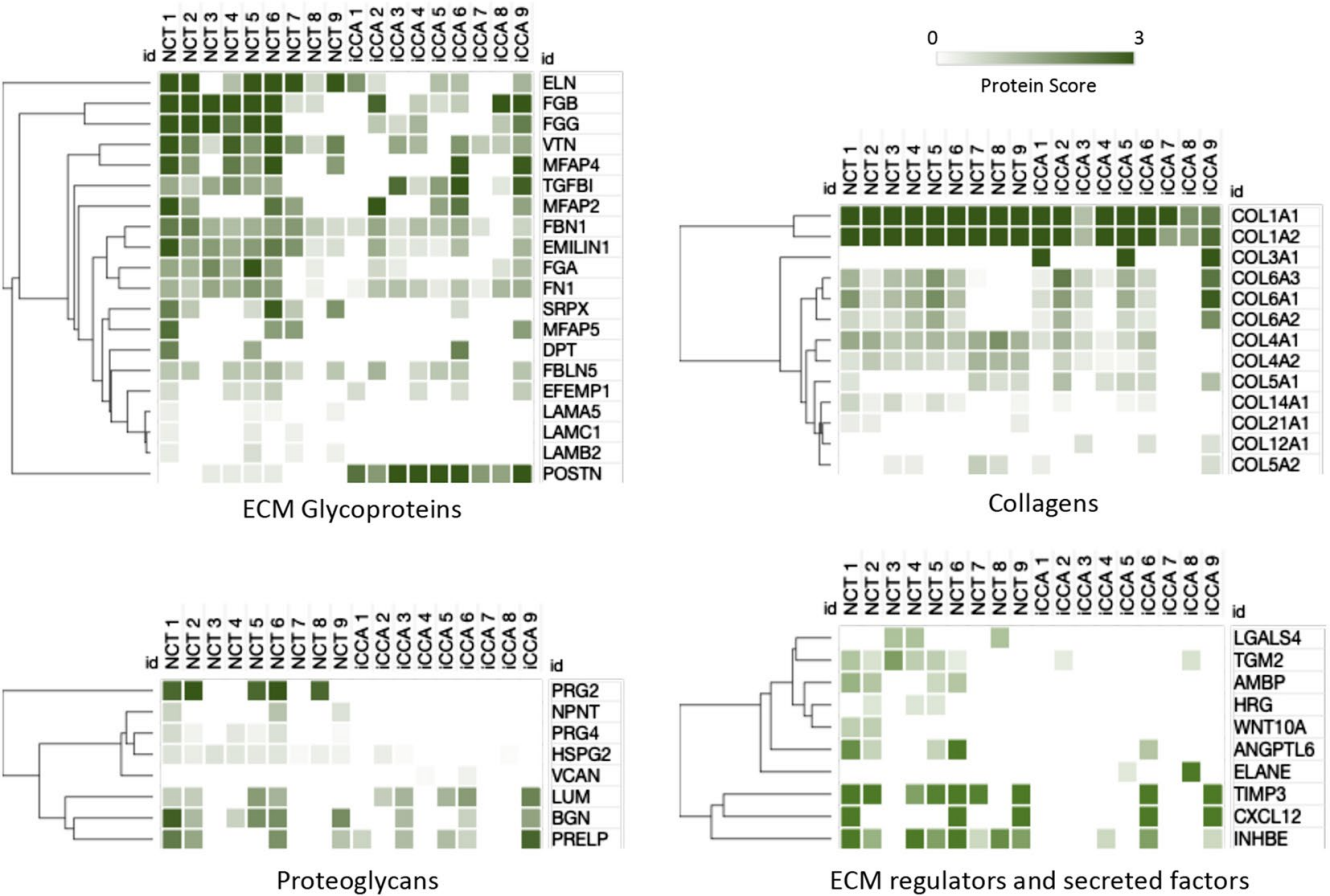
Semi-quantitative analysis of ECM proteome profiles in NCT and iCCA samples. Protein scores from matrisome data sets were calculated and analysed by heat map analysis. Two-dimensional hierarchical clustering was performed with a weighted Euclidean method (Morpheus, https://software.broadinstitute.org/morpheus). Each vertical column represents an individual sample and each horizontal row an individual protein. The color scale indicates the magnitude of protein scores

Matrisome analysis unveiled specific ECM proteome profiles between the iCAA and NCT samples (Fig. 2 and Additional file 1). Overall, the ECM of iCCA samples was generally composed by fewer ECM protein components in respect to the liver parenchyma, particularly in terms of glycoproteins, proteoglycans, regulators and secreted factors. As expected, reduced levels of TIMP3 and elastin (ELN), the major components of elastic fibers, were found in iCCA proteome profiles, while high levels of POSTN were found in all the cancer samples. These data indicated the robustness of proteomics observations. Among the down-expressed proteins in the iCCA samples, several basement membrane proteins have been identified: laminin α5 (LAMA5), laminin β2 (LAMB2), laminin γ1 (LAMC1), collagen type 4 alpha-1 chain (COL4A1), collagen type 4 alpha-2 chain (COL4A2) and basement membrane-specific heparan sulfate proteoglycan (HSPG2). These data indicate the disarrangement of the basement membrane in the iCCA tumorigenesis. Interestingly, the three fibrinogen chains (FGA, FGB, FGG) and tissue transglutaminase (TGM2), known to be upregulated in other desmoplastic tumors [20, 21], were found poorly expressed in iCCA. The most noteworthy changes in the iCCA ECM were found in the low levels of Inhibin beta E chain (INHBE), proteoglycan 2 (PRG2) and 4 (PRG4), and in the expression of collagen type 3 alpha-1 chain (COL3A1) and collagen type 12 alpha-1 chain (COL12A1). Overall, these data provide an iCCA matrisome profiling, where down-expressed and overexpressed proteins may serve as a list of possible candidates for further investigation.

### Validation of COL3A1, PRG2 e PRG4 expression levels in iCCA and NCT tissues

**Fig. 3:**
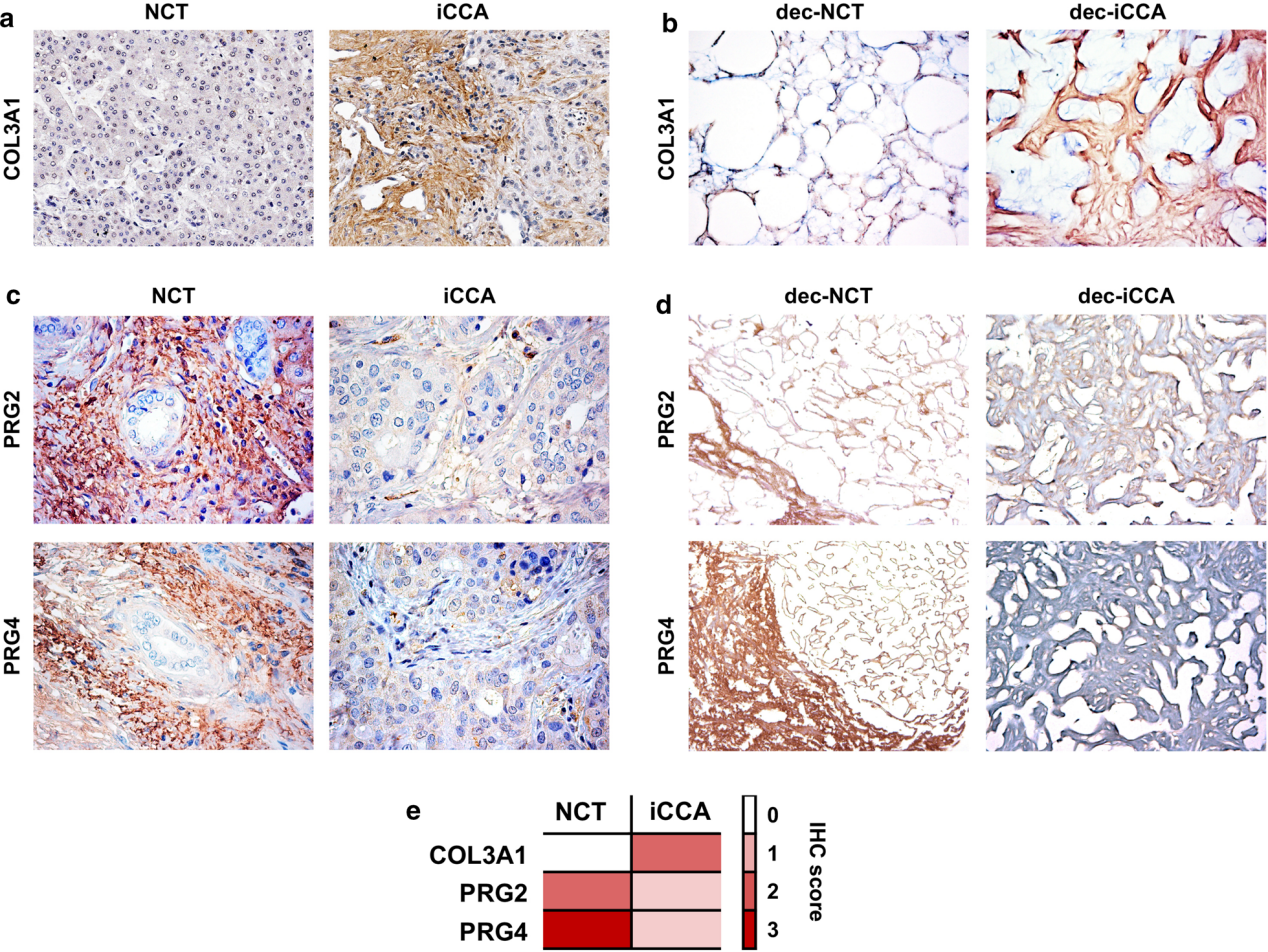
COL3A1, PRG2 and PRG4 expression in NCT and iCCA tissues (N = 9). Representative images of immunohistochemistry for COL3A1, PRG2 and PRG4 in NCT and iCCA histological sections. To increase the detection levels, the IHC analysis of COL3A1 (**a**, **b**) and proteoglycans (**c**, **d**) has been performed on intact and decellularized (dec) tissues. Original magnification = ×20 (**a**, **d**) and ×40 (**b**, **c**). Semi-Quantitative data (Median) are shown in the heat map (**e**) according to the following scoring system: 0 = < 10%; 1 = 10–30%; 2 = 31–50%; 3 = > 50%. At least 5 non-overlapping field at ×20 were analysed

Type III collagen, a fibrillar-forming collagen made up of three copies of COL3A1 chain, is expressed in many connective tissues [22]. Since COL3A1 was found at high levels in iCCA, its expression was further confirmed on NCT and iCCA tissues by immunohistochemistry both in intact and decellularized tissues. As reported in Fig. 3, increased levels of COL3A1 were observed in the iCCA (mean ± standard deviation = 1.8 ± 1.4) samples compared to paired NCT samples (mean ± standard deviation = 0.2 ± 0.4; p < 0.05). Additional file 2 shows that 3/9 iCCA samples expressed very low to negligible levels of COL3A1 (semiquantitative score = 0) and that 6/9 iCCA samples have higher COL3A1 expression compared to NCT.

PRG2 and PRG4 are proteoglycans mainly expressed in eosinophil granules, liver and in the synovial fluid [23, 24]. Since PRG2 plays a tumor suppressor function, and PRG4 shows an anti-inflammatory role [25, 26], the downregulation of these proteoglycans in the cancerous ECM have been additional investigated by IHC analysis in paired samples (Fig. 3), and confirm the results obtained from the proteomics analysis.

### Expression of COL3A1 in the iCCA desmoplastic extracellular space

**Fig. 4:**
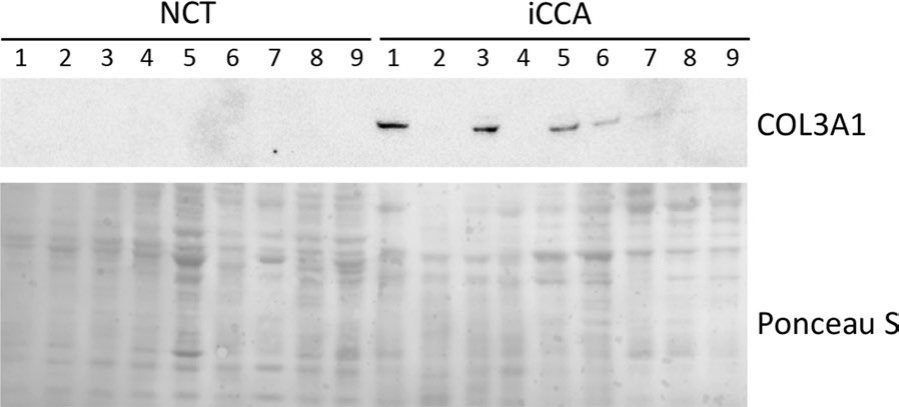
Representative western blot analysis for COL3A1 in NCT and iCCA extracellular spaces. Total protein staining by Ponceau S on the nitrocellulose membrane is shown. See “Methods” for details

Desmoplastic reaction in iCCA requires the ECM remodelling which results in release of proteins related to both synthesis and degradation of the matrix [5, 6]. Therefore, the released amount of COL3A1 chains in the extracellular space was also investigated. To this aim, protein flushed out from NCT and iCCA specimens were individually collected and analysed by western blotting analysis. Interestingly, COL3A1 was detected in almost all tumor samples only (Fig. 4). In the frame of type III collagen structure, this data suggests that not all the COL3A1 chains are wrapped to form fibrils and fibers of the tumor ECM scaffold.

### Type III collagen promotes migration of iCCA primary cells

**Fig. 5:**
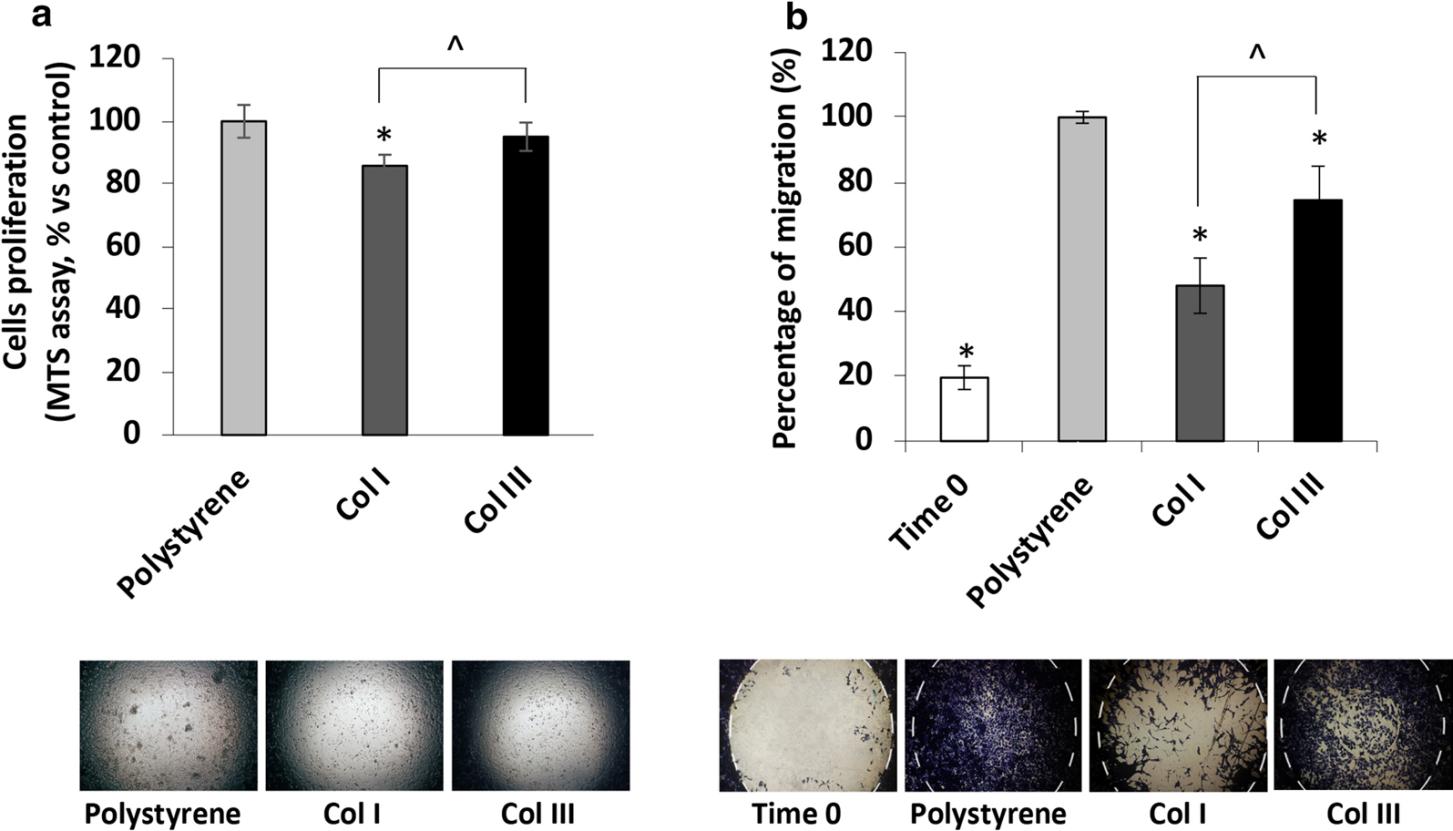
Effect of type I (Col I) and type III (Col III) collagens on proliferation and migration of primary human iCCA cell cultures. **a** Cell proliferation was evaluated by MTS assay after 72 h of treatment and expressed as percentage value compared to Polystyrene used as control (100%). Representative phase contrast photographs are shown. **b** Cell migration was evaluated at 7 days of cultivation by calculating the percentage of covered area (percentage of migration, %). Representative phase contrast photographs at day 7 are shown; dotted white lines indicate the initial area available to cells at time 0. All data represent mean ± SD of N = 5 independent experiments. *p < 0.05 vs. Polystyrene (Control); ^p < 0.05 Col I vs. Col III

Among the significant over-expressed proteins identified in iCCA (i.e.: POSTN, COL3A1, COL12A1), the overexpression of periostin has been extensively demonstrated to promote cholangiocarcinogenesis [9, 10, 27, 28]. Since it has been reported that, in various invasive cancers, the overexpression of COL3A1 promotes tumor progression and confers poor prognosis, we therefore investigated the possible effects of COL3A1 in modifying iCCA cells behaviour. To this end, we made use of a primary cell culture, obtained from iCCA patients submitted to surgical resection, that has been extensively characterized as an iCCA in vitro model in physiopathology studies [16–18]. Then, proliferation and migration activities were evaluated by culturing the iCCA primary cells either on type I or type III collagen-coated wells. The proliferative capability of iCCA primary cells cultured on type III collagen-coated wells was increased compared with those coated with type I collagen (Fig. 5a). Interestingly, after 7 days of culturing, the cell migration on type III collagen was significantly accelerated in respect to type I collagen (Fig. 5b).

### Type III collagen alignment in iCCA

**Fig. 6:**
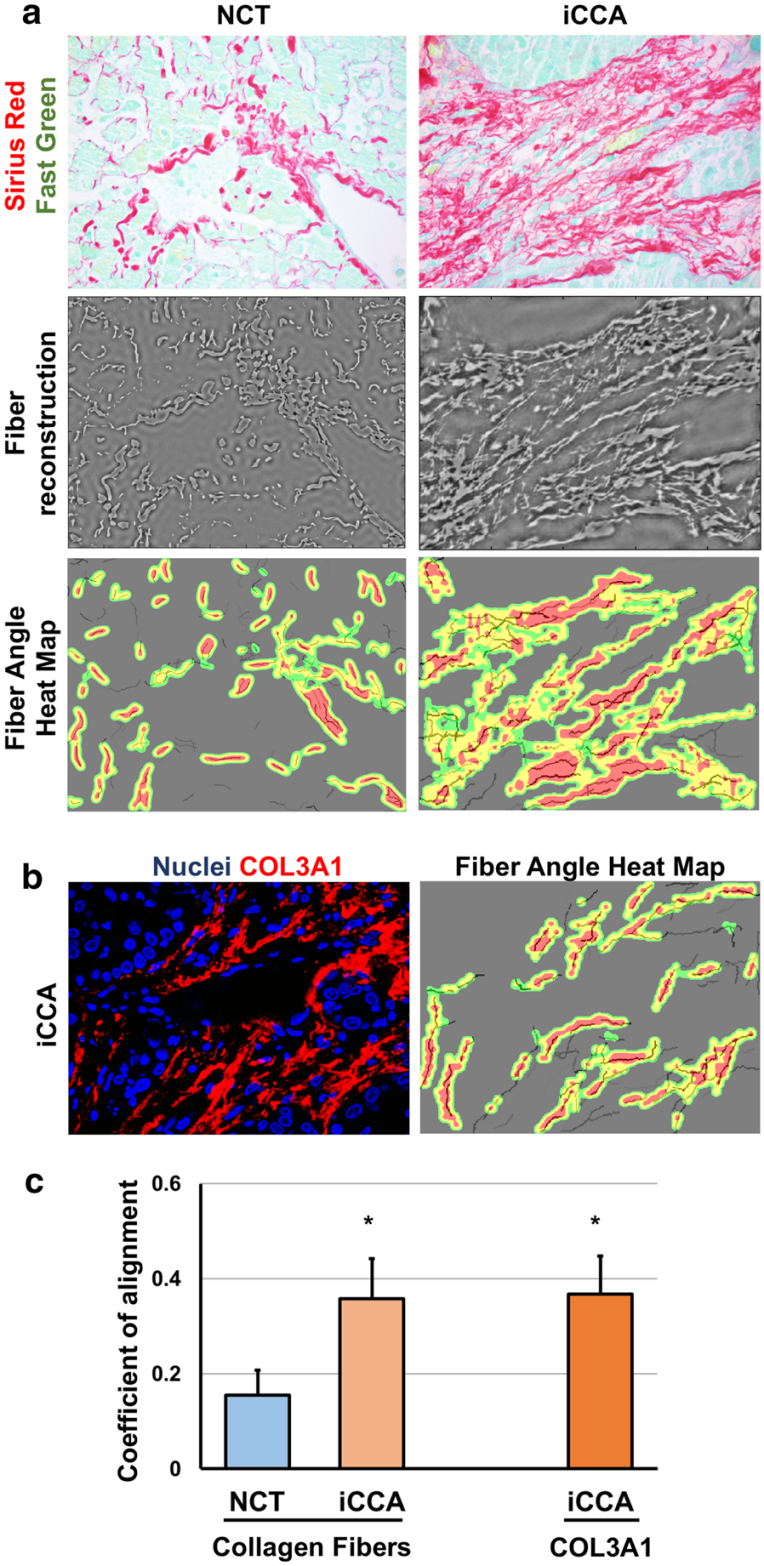
Analysis of collagen alignment in iCCA and NCT samples (N = 9). **a** The collagen fiber organization was studied on Sirius Red/Fast Green stained sections (upper panel). The fibers were identified and reconstructed by the ct-FIRE software package (middle panel). The coefficient of alignment of total collagen fibers was calculated by the CurveAlign software package and visualized by heat maps using red color to indicate well aligned fiber region (lower panel). **b** COL3A1 fibers were identified by immunofluorescence (left image); immunofluorescence images were processed with ct-FIRE and CurveAlign software packages; the coefficient of alignment is visualized by the heat map using red color to indicate well aligned fiber region (right image). **c** Histogram shows that iCCA samples have a relatively higher alignment coefficient for their collagen organization in respect to NCT samples with COL3A1 as a component of iCCA-induced aligned collagen. Coefficient of alignment ranges from completely random fiber orientation, 0.0, to completely aligned fiber orientation, 1.0; *p < 0.05 versus NCT. Analyses were performed on 5 non-overlapping fields at ×20 magnification per sample

Tumor progression is accompanied by collagen fiber reorganization, called Tumor-Associated Collagen Signature (TACS) [29, 30]. Initially, collagen fibers acquires a dense, wavy and curly architecture (TACS-1), subsequently it appears straight and aligned parallel to the tumor boundary (TACS-2), and, in the last stages of tumor progression, perpendicular to it (TACS-3). Importantly, it has been demonstrated that aligned collagen fibers are crucial for cancer cell migration and invasion [30, 31]. Here, we analysed the collagen fiber organization in the frame of iCCA. The NCT tissues presented curly/wavy, randomly organized collagen fibers while the iCCA samples showed straightened/aligned collagen fiber architecture (Fig. 6a). This implied a relatively higher coefficient of alignment for the iCCA total collagen fiber organization in respect to NCT samples (Fig. 6b). Interestingly, the coefficient of alignment specifically measured for COL3A1 showed a similar score to the total collagen fibers (Fig. 6b, c), thus suggesting the involvement of the type III collagen in the ECM re-organization during tumor progression.

## Discussion

Tumor progression and dissemination are critically supported by the ECM of tumor microenvironment. Therefore, it is not surprising that, in the last years, proteomics studies investigating the composition and the role of the ECM in many cancers types have considerably increased [32–36]. However, although evidences on the role of some ECM components in iCCA progression have been provided, a comprehensive cancer matrisome characterization had not yet been attempted.

This study is the first characterization of the ECM proteome profile in iCCA microenvironment. Our analysis allowed unveiling a unique tumor-associated ECM fiber composition, thus indicating that a substantial ECM architecture reorganization occurred during cancer development. Taken together, proteomics and histological data showed that the iCCA ECM is mainly composed by high levels of fibrillar collagens (i.e.: type I, III and the fibril associated collagen XII) and periostin. The almost total absence of elastic fibers and the low expression of proteoglycans, ECM glycoproteins and regulators clearly explain the stiff, poorly hydrated and highly dysregulated nature of the iCCA desmoplastic stroma. Down regulation of reticular fibers, as well as of laminins, network-forming type IV collagen and the basement membrane-specific heparan sulfate proteoglycan (HSPG2), demonstrated the disarrangement of basement membrane, leading to a disorganized network. ECM bio-mechanical properties, particularly the grade of elasticity, influence cancer cell invasion and metastasis [37]. Particularly, ECM stiffness sustains tumor progression by displacing the host tissue and promotes cancer cell invasion towards surrounding tissues by mechanotransduction [37]. Tumor progression is further favoured by the disaggregation of the basement membrane, which in non-diseased tissue acts as a physical barrier against cell migration and invasion [38]. Therefore, the iCCA-induced ECM fibers profile emerged from this proteomic study is consistent with a deregulated matrix whose features support cancer cells spreading.

The finding of altered ECM fiber composition prompted us to search for proteins playing a role in iCCA aggressiveness and responsible for iCCA desmoplastic reaction. By proteomics approach, we observed that ECM components known to have tumor suppressor functions and anti-proliferative effects (i.e.: INHBE and PRG2) were found strongly downregulated in iCCA [25, 39]. Furthermore, the downregulation of angiopoietin-related protein 6 (ANGPTL6) and the sushi repeat-containing protein 2 (SRPX2) shed light on the molecular basis of hypovascular nature of iCCA. Focusing attention on the ECM proteins found overexpressed in the iCCA with respect to NCT, COL3A1 appeared to play a primary role in promoting different tumor pathological features. Although type III collagen role in ECM organization and function is still poorly, recent evidences indicated a clear role of COL3A1 in promoting the progression of invasive solid cancers and its involvement in the tumorigenesis of many cancers type [40–42]. Furthermore, COL3A1 has been demonstrated to be a target of miR-29a/b, whose downregulation is responsible for the increased invasiveness of lung cancer [43]. Interestingly, in renal cell carcinoma, let-7d miRNA suppresses growth, metastasis, and tumor macrophage infiltration by directly targeting COL3A1 [41]. Our data confirmed an involvement of COL3A1 in supporting primary iCCA cells migration.

This finding prompted us to investigate the biomechanical properties of COL3A1 in iCCA by analysing the type III collagen structural organization in tumor stroma. Notably, for the first time, we described a straight and aligned collagen architecture in iCCA. As for the breast carcinoma [44], this finding suggests that the aligned collagen pattern may be a suitable hallmark of iCCA formation and progression. Interestingly, we demonstrated that COL3A1 is involved in the iCCA-induced aligned collagen. Since type III collagen is the main component of reticular fibers, this finding suggests that COL3A1 expression in iCCA maybe hijacked from reticular to aligned fibers formation. Aligned collagen has been characterized to be a binary track providing contact guidance cues for cancer cell migration and invasion [45]. In this context, our findings on the involvement of type III collagen in tumor-induced collagen alignment, as well as in promoting iCCA cells migration, urge to further investigate the COL3A1 as potential molecular target. In particular, before any eventual therapeutic relevance, it is required to overcome two important limitations of this study: (i) while supported by in vitro observations, the role of COL3A1 in cancer cell spreading should be extended on animal models of cholangiocarcinoma [46]; (ii) type III collagen alignment should be integrated and interpreted in a larger cohort of iCCA patients at different stages of the disease.

ECM leakage proteins released into the extracellular medium may reflect the state of the cancer onset and progression. Additionally, we demonstrated that COL3A1 chains are expressed also in the liquid extracellular space. Therefore, this finding may have implications for the non-invasive detection of the disease. In this direction, further investigations on sera of iCCA patients are needed.

Overall, the iCCA matrisome profile suggested that the paracrine communication between CAFs and iCCA cells that fuels cancer aggressiveness may reside only a few components of the ECM.

## Conclusions

In summary, this study unveils the ECM structural and molecular composition of the iCCA microenvironment. We believe that our findings contribute to the understanding of the molecular basis underlying the iCCA-induced desmoplastic reaction and suggests COL3A1 as a promising molecular target in hampering tumor expansion and metastatic dissemination.

## Supplementary information

**Additional file 1.** Mass spectrometry data reports for iCCA and NCT samples.

**Additional file 2.** Immunohistochemistry (IHC) semi-quantitative score for COL3A1 expression in samples obtained from each patient.

## Data Availability

All identified proteins have been uploaded as additional files in this article. The raw data of mass spectrum will be available from the corresponding author on reasonable request.

## Abbreviations

iCCA: intrahepatic cholangiocarcinoma
α-SMA: α-smooth muscle actin
CAFs: cancer associated fibroblasts
ECM: extracellular matrix
POSTN: periostin
CXCL12: stromal cell-derived factor-1
MMP: metalloproteinase
NCT: non-cancerous liver tissue
COL: collagen
DB: decellularization buffer
DTT: dithiothreitol
IAM: iodoacetamide
NanoLC: nano liquid chromatography
ACN: acetonitrile
MALDI-TOF/TOF: matrix assisted laser desorption ionization-time of flight/time of flight
CHCA: a-cyano-4-hydroxycinnamic acid
RSLC: rapid separation liquid chromatography
H&E: hematoxylin–eosin
SR: Sirius Red
PRG: proteoglycan
PBS: phosphate buffered saline
RT: room temperature
SQ: semi-quantitative
ELN: elastin
LAM: laminin
HSPG2: heparan sulfate proteoglycan
TGM2: tissue transglutaminase
TACS: tumor associated collagen features
ANGPTL6: angiopoietin-related protein 6
SRPX2: sushi repeat-containing protein 2
