# Supplementary material for: Matrisome analysis of intrahepatic cholangiocarcinoma unveils a peculiar cancer-associated extracellular matrix structure
Source: Clin Proteomics. 2019 Oct 30;16:37. doi: 10.1186/s12014-019-9257-x (PMC6821022; doi:10.1186/s12014-019-9257-x)
Supplement: Supplementary file 2 — Additional file 2. Immunohistochemistry (IHC) semi-quantitative score for COL3A1 expression in samples obtained from each patient. [file 12014_2019_9257_MOESM2_ESM.pdf]

Additional file 2

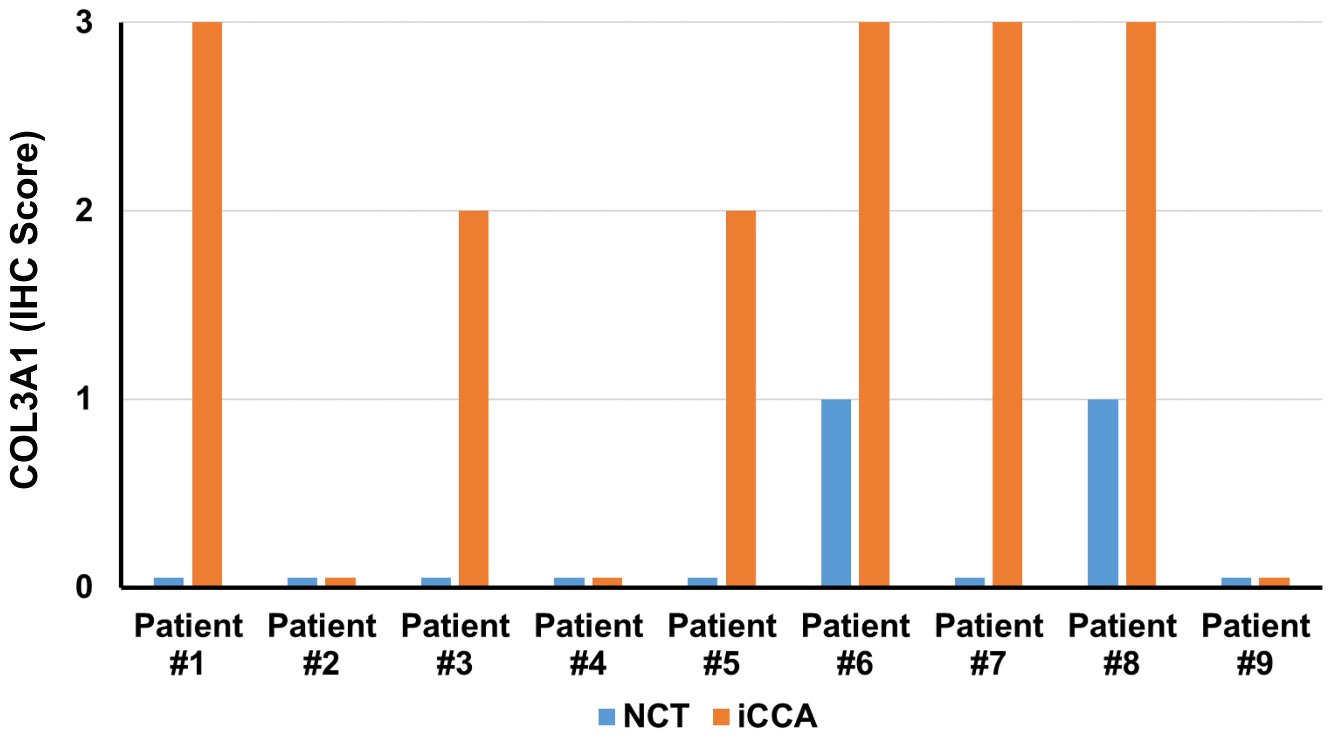

Immunohistochemistry (IHC) semi-quantitative score for COL3A1 expression in samples obtained from each patient. iCCA: intrahepatic cholangiocarcinoma; NCT: non cancerous liver tissue
